# Patient participation in decisions about disease modifying anti-rheumatic drugs: a cross-sectional survey

**DOI:** 10.1186/1471-2474-15-333

**Published:** 2014-10-04

**Authors:** Ingrid Nota, Constance HC Drossaert, Erik Taal, Harald E Vonkeman, Mart AFJ van de Laar

**Affiliations:** Department of Psychology, Health and Technology, University of Twente, PO Box 217, Enschede, 7500AE The Netherlands; Department of Rheumatology and Clinical Immunology, Medisch Spectrum Twente, PO Box 50 000, Enschede, 7500 KA The Netherlands

**Keywords:** Shared decision-making, Patient involvement, Patient participation, Arthritis, Disease modifying anti-rheumatic drugs

## Abstract

**Background:**

Involvement of patients in decision-making about medication is currently being advocated. This study examined (the concordance between) inflammatory arthritis patients’ preferred and perceived involvement in decision-making in general, and in four specific decisions about Disease-Modifying Anti-Rheumatic Drugs (DMARDs). Furthermore, this study examined how patients’ involvement is related to satisfaction about decision-making and which factors are related to preferred roles, perceived roles and concordance.

**Methods:**

Using a cross-sectional survey, 894 patients diagnosed with Rheumatoid Arthritis, Psoriatic Arthritis or Ankylosing Spondylitis were sent a questionnaire which focused on medical decisions in general and on four specific decisions: (a) starting with a traditional DMARD; (b) starting to inject methotrexate; (c) starting a biological DMARD; and (d) decreasing or stopping a DMARD. For each decision preferred and perceived involvement in decision-making was assessed using the Control Preference Scale. Concordance was calculated by subtracting the scores for perceived role from scores for the preferred role. Furthermore, satisfaction with the decision process and socio-demographic, health-related, patient-related and physician-related variables were assessed.

**Results:**

The response rate was 58%. For all decisions, most patients (59%-63%) preferred Shared Decision-Making (SDM). SDM was perceived frequently (26%-55%) and patients’ preferences were met in 54% of the respondents. Yet, in some specific decisions, 26% to 54% of patients would have liked more participation. Perceiving less participation then preferred was associated with less satisfaction with the decision-process, but perceiving more participation than preferred was not. Our results did not reveal any meaningful models to predict preferred or perceived participation in decision-making in general or with reference to specific decisions about DMARDs.

**Conclusions:**

Most arthritis patients prefer to be involved in decisions about their medication and SDM is perceived frequently. Yet, in some specific decisions patient participation can be further improved. Patients especially prefer more participation in decision-making regarding starting a first traditional DMARD, which occurs most commonly in newly diagnosed patients. Whereas perceiving too little participation was associated with decreased satisfaction, perceiving too much participation was not. Therefore, rheumatologists should urge patients to participate in every medical decision.

## Background

Medication use is central to the management of rheumatic diseases and medication adherence is essential for the success of the treatment. Traditionally, decisions about medication have been viewed from a paternalistic perspective where the prescriber makes decisions based on medical knowledge and the patient either complies or does not comply with the prescribed regime (also defined as clinician-led decision-making).

Currently, involvement of patients in decision-making about medication is being advocated. Patient involvement in decision-making is considered beneficial for various reasons. First, the patient’s agreement with the choice of treatment is important since the patient’s cooperation in carrying out the treatment is essential [1]. Secondly, Shared Decision-Making (SDM) is assumed to lead to improvement in health outcomes, such as health status, self-management, adherence, coping behavior and satisfaction with care [2–5], especially in chronic diseases [6]. Finally, patients have the right to self-determination and should thereby be empowered by information about their diagnosis, treatment options and prognosis to make treatment decisions that correspond with their preferences and values [7].

Whereas patient participation is considered to be important and beneficial, SDM can be difficult to achieve for both doctors and patients. Doctors are often reluctant or unprepared to involve patients in medical decisions [8, 9]. Some barriers mentioned by doctors are lack of time and low confidence in their ability to communicate risks effectively [10]. Patients also experience barriers, such as unawareness of having a choice, low confidence to participate, a belief of having a lack of knowledge and uncertainty about which questions to ask [11, 12].

Furthermore, not all patients want to be actively involved in medical decision-making. Results of previous studies concerning patients’ preferences regarding participation in treatment decisions show high variability [13–17], including the field of rheumatology [18–21]. Although, there is extensive literature that has examined factors (socio-demographics, health-related, patient-related and physician-related) that predict patients’ preferences regarding involvement, results are inconclusive and it remains difficult to explain or predict patient preferences [13, 19, 21–26]. Garfield, Francis and Smith [25] suggest that preference regarding involvement might vary per type of decision. Moreover, role preference may change over time and change as health status changes [11, 13, 17, 19, 23, 27]. Thus, to pursue concordance between patients’ preferred and actual role in decision-making, it is essential to study patients’ preferences regarding involvement and to discriminate between specific decisions.

Compared to decisions in acute care, decisions in chronic care are more likely to need an active patient role in executing the decision [28]. In rheumatology, decisions about medication reoccur during the process of the disease and are likely to be revised and reversed. However, the latter does not make it easier to make a decision. Treatment decisions have become increasingly complex due to the many new available Disease-Modifying Anti-Rheumatic Drugs (DMARDs). These drugs vary with respect to approximate time to benefit, side effects and risks, dosage, and route of administration.

Four specific decisions regarding DMARDs are particularly relevant: (a) starting with traditional DMARDs; (b) starting to inject methotrexate; (c) starting a biological DMARD; and (d) decreasing or stopping a DMARD. Guidelines strongly recommend early intervention with a traditional DMARD [29–31]. The recommended traditional DMARD of first choice is methotrexate, which can be administered orally or by subcutaneous injection. In case of intolerance or disfavour for methotrexate, other traditional DMARDs are good alternatives. In the Netherlands, therapy with a biological DMARD can only be prescribed to patients with at least moderate disease activity and in whom treatment with at least 2 traditional DMARDs has failed. The decision to decrease or stop medication occurs when the disease is in remission or when side effects are presented.

Although it seems the management of inflammatory arthritis is strongly protocolled, involving patients in decision-making about DMARDs is important, as some of the DMARDs can have serious side effects and the route of administration (orally, subcutaneous injection or intravenous injection) may have a large impact on patients’ daily lives. Thus, to choose the best treatment is a process concerning clinical aspects, but also patients’ preferences need to be considered. After all, these decisions require an active patient role in carrying out the decision and adherence is essential for the success of the treatment.

More knowledge about inflammatory arthritis patients’ preferred level of involvement could lead to rheumatologists and other caregivers anticipating on this and make it easier to pursue the preferred level of patient involvement. We expect patients to be more satisfied with the decision-process if concordance is reached between the preferred and perceived level of participation. Whereas a few studies have examined inflammatory arthritis patients’ preferred and/or perceived role in medical decision-making in general, to the best of our knowledge there is no data comparing patients’ preferred and perceived role in specific decisions. Therefore, this study focused on inflammatory arthritis patients’ preferred and perceived participation in various decisions related to the use of DMARDs. We studied the concordance between preferred and perceived roles and the perceived satisfaction about the decision process. Furthermore, we examined which factors (socio-demographic, health-related, patient-related and physician-related) are associated with the preferred and perceived roles.

## Methods

### Sample and setting

We focused our cross-sectional survey on patients with rheumatic diseases who were likely to use DMARDs: patients diagnosed with Rheumatoid Arthritis (RA), Psoriatic Arthritis (PsA) or Ankylosing Spondylitis (AS). Patients were recruited from two hospitals in the Netherlands: Medisch Spectrum Twente (MST) and Ziekenhuisgroep Twente (ZGT). Patients were selected with use of the electronic hospital records. First, a random sample of 965 patients (500 from MST, 465 from ZGT) who met the following criteria was selected: (a) consulted their rheumatologist in the past year; and (b) were diagnosed with RA, PsA or AS. The list of selected patients was then discussed with the treating rheumatologist. Based upon this, 71 patients were excluded because either the patient (1) was deceased, (2) had an incorrect diagnosis registered in the electronic hospital record, or (3) was not able to complete a Dutch written questionnaire (subjective interpretation by the rheumatologist). In total 894 eligible patients were sent a questionnaire by mail, accompanied by a letter of invitation from their rheumatologist and an informed consent form. The patients were asked to return the filled out questionnaires and the informed consent form to the University of Twente using a prepaid envelope. After three weeks a reminder was sent.

The study did not need approval of the ethical review board according to the Dutch Medical Research Involving Human Subjects Act (WMO); only (non-intervention) studies with a high burden for patients have to be reviewed.

### Measures

Standardized scales were used as much as possible. If there was no Dutch scale available, scales were translated using the forward-backward procedure [32].

The questionnaire contained 65 questions and focused on medical decisions in general and on four specific decisions: (a) starting with a traditional DMARD; (b) starting to inject methotrexate; (c) starting a biological DMARD; and (d) decreasing or stopping medication. To make it easier for patients to remember the decisions addressed in the questionnaire, a short description of each decision was given including purpose, route of administration and generic and brand medicine names (see Table 1). For each decision patients were asked (1) whether they had ever faced the decision, and if relevant, patients were asked to think of the first time they faced the decision. Then they were asked (2) what role they had perceived, (3) what the outcome of the decision had been (e.g. starting or not starting with the suggested medication), and (4) if they were satisfied with the decision-making process. Subsequently, all patients (including patients who had never faced the decision) were asked what role they preferred to have. Furthermore, socio-demographic, health-related, physician-related and patient-related variables were questioned.

**Table 1:** **Description of decisions as provided in questionnaire (translated from Dutch)**

Decision	Description
**Starting traditional anti-rheumatic drugs.**	The following questions concern starting traditional anti-rheumatic drugs, also called traditional DMARDs. These drugs can reduce joint damage. They decrease disease activity: they ease pain and rigor and on the long term prevent further joint damage.
	*Examples: methotrexate (Emthexate* ***®*** *, Ledertrexate* ***®*** *), sulfasalazine (Salazopyrine* ***®***); *go* *l* *d (Tauredo* ***®*** *, Ridaura* ***®*** *), hydroxychloroquine (Plaquenil* ***®*** *), penicillamine (Gerodyl* ***®*** *), azathioprine (Imuran* ***®*** *), ciclosporine (Neoral* ***®*** *) and leflunomide (Arava* ***®*** *).*
**Starting to inject.**	Medication can be administered in various ways. Most drugs are administered orally as tablets. Another way is by subcutaneous injection. Methotrexate (Emthexate®, Ledertrexate®) is available as tablet, but can also be administered by subcutaneous injection. The following questions concern starting subcutaneous methotrexate injections.
	*Beware: these questions only concern methotrexate and not other drugs that may be administered by subcutaneous injection.*
**Starting biologic anti-rheumatic drugs.**	The following questions concern starting biologic anti-rheumatic drugs, also called biologic DMARDs. Biologic DMARDs are administered by subcutaneous injection or directly into a vein. Biologic DMARDs aim to reduce arthritis by inhibiting mediators of inflammation, such as TNF and Interleukine-1.
	*Examples: Adalimumab (Humira* **®** *), Etanercept (Enbrel* **®** *), Infliximab (Remicade* **®** *), Anakinra (Kineret* **®** *).*
**Decreasing or stopping anti-rheumatic drugs.**	For various reasons medication can be decreased or even stopped. This may be due to side effects or because you are doing so well that the dosage may be decreased.
	The following questions concern decreasing or stopping anti-rheumatic drugs.
	*Beware: these questions only concern your anti-rheumatic drugs and not pain medication or other drugs.*

#### Preferred and perceived participation and concordance

Preferred and perceived roles in medical decision-making were assessed with the ‘Control Preference Scale’ (CPS) [33] adapted by Garfield, et al. [19]. Questions about the perceived role started with “In your opinion, who decided to …”; questions about the preferred role started with “If you are informed about the benefits and risks, who should finally decide about …”. Response categories were: “The rheumatologist” (1), “Mostly the rheumatologist” (2), “The rheumatologist and me together” (3), “Mostly me” (4), and “Me alone” (5). Further, we recalculated the CPS scores to 3 levels: doctor (1–2), shared (3) and patient (4–5), as validated by Degner [33]. Concordance was calculated by subtracting the original perceived CPS scores from the original preferred CPS scores. The results ranged from −4 to 4 and were then coded into 3 levels: too little participation (<0), enough participation (0) and too much participation (>0).

#### Satisfaction

For each specific decision, satisfaction with the decision-making process was assessed with one item ‘How satisfied are you about how this decision was made?’ using a five-point Likert scale (very unsatisfied (1) – very satisfied (5)).

Socio-demographic variables included sex, age, marital status, education (low, medium, high), income (low, medium and high) and work status (employed vs. unemployed, volunteer, student, retired, or homemaker).

Health related variables included diagnosis (RA, PsA or AS), time since diagnosis (<1 year, 1–5 years, 5–10 years, or >10 years) and health-related quality of life. Health-related quality of life was assessed with the SF-12, version 2 [34]. Standardized scores were calculated for the physical and mental well-being varying from 0 (poor well-being) to 100 (excellent well-being), with a mean of 50 and a standard deviation of 10 in the general population of the United States [34].

#### Patient-related variables

Need for information was assessed with a subscale of the Autonomy Preference Index (API) [23]. The API consists of 8 items and patients respond on a five-point Likert scale. Response choices range from 0 (strongly disagree) to 4 (strongly agree). Sum scores were linearly adjusted to range from 0 (no need for information) to 100 (strongest possible need for information). Internal consistency was adequate (Cronbach’s α = 0.66).

Patients' self-efficacy in obtaining medical information and attention to their medical concerns by physicians was assessed using the ‘Perceived Efficacy In Patient-Physician Interaction’ (PEPPI) scale [35]. The PEPPI consists of 10 items, each beginning with ‘How confident are you in your ability to…’ and using response options 1 (not at all confident) to 5 (very confident). Scores on the 10 items were added for each patient to acquire a total score, with higher scores indicating more self-efficacy. Internal consistency was good (Cronbach’s α = 0.91).

#### Physician-related variables

Characteristics of consultations with the rheumatologist included three variables: 1) frequency of visits in the last year (once a year, 2–4 times a year, more than 4 times a year), 2) having a regular rheumatologist (‘How often do you consult the same rheumatologist’) using a five-point Likert scale (always – never) and 3) duration of the relationship with the rheumatologist (in years).

Perceived trust in the physician and emotional support from the physician were assessed with 2 subscales of the ‘Cologne Preference Questionnaire’ (CPQ) [36, 37]. Perceived trust in the physician was measured with 3 items. Patients’ evaluation of emotional support was assessed with 4 items. Response choices range from 1 (strongly disagree) to 4 (strongly agree). Scale scores were computed for each scale by the mean of the items. Both scales range from 1 to 4 with a lower score indicating lower trust or lower emotional support. Internal consistency of both scales were good (Cronbach’s α = 0.93 and α = 0.85, respectively).

Prior to inclusion, we performed a pilot test among patients (n = 8) to assess the readability and acceptability of time to complete the questionnaire. The test showed that the questionnaire took about 20–25 minutes to complete, which was acceptable according to the participants. Minor textual adjustments were made following the results of the pilot test.

### Statistical analysis

To detect differences in the distributions of (concordance between) preferred and perceived participation across decisions, chi-square tests were performed. Chi-square tests were also used to detect differences in the distribution of preferred participation between respondents who had faced the decision versus respondents who had not faced the decision.

The Kruskal Wallis test was used to compare differences in satisfaction between groups with different levels of perceived participation and with different levels of concordance. Next, a post hoc Mann–Whitney U test with Bonferroni correction was used to test which groups were significantly different from each other.

To examine which factors are associated with preferred role and perceived role we performed multivariate binary logistic regression analyses. We predicted the preference for and perception of shared decision-making compared to clinician-led decision-making. The relationship with patient-led decision-making has not been analysed because of the too small numbers of patients preferring or perceiving this (frequencies ranging from 6 to 76, depending on the type of decision) relative to the number of predictors (n = 13). We included the following predictors: age, sex, education, employment, years since diagnosis, physical and mental well-being, self-efficacy in patient-provider interaction, need for information, frequency of visits in the last year, duration of relationship with the rheumatologist, trust in physician and emotional support of physician.

## Results

### Patient characteristics

We received 519 completed questionnaires (response rate 58%). The sample of respondents was heterogeneous in regard to socio-demographic and health related variables (Table 2). The mean score for physical wellbeing was 39, somewhat lower than that of the US general population (50), but similar to that of the US RA population [34]. The mean score for mental wellbeing was similar to that in the general population. Most respondents visited their rheumatologist 2–4 times per year (n = 344; 67.5%) and saw the same rheumatologist at almost every visit (n = 501; 97%).

**Table 2:** Demographic, health-related, physician-related and patient-related characteristics (n = 519)*

Variables	Categories	Value
**Socio-demographic variables**		
Age, years		56 ± 12
Women, no. (%)		285 (59)
Married/living with a partner, no. (%)		391 (82)
Education, no. (%)	Low (<12 years)	155 (33)
	Medium (12 – 16 years)	220 (47)
	High (>16 years)	94 (20)
Family income, no. (%)	Low (< €28.500/year)	114 (31)
	Medium (€28.500 - €34.000/year)	112 (31)
	High (> €34.000/year)	139 (38)
Fulltime and part time employed, no. (%)		198 (45)
**Health-related variables**		
Diagnosis (n,%)	Rheumatoid Arthritis	307 (63)
	Psoriatic Arthritis	120 (25)
	Ankylosing Spondylitis	58 (12)
Years since diagnosis, no. (%)	<1	19 (5)
	1–5	82 (21)
	6–10	159 (40)
	>10	139 (35)
Well-being (SF-12) (range 0–100)	Physical	39 ± 10
	Mental	49 ± 10
**Patient-related**		
Self-efficacy in patient-provider interaction (PEPPI) (range 10–50)		39.9 ± 4.2
Need for information (API) (range 0–100)		71.7 ± 10.3
**Physician-related variables**		
Frequency of visits in the last year, no. (%)	once a year	75 (14.7)
	2–4 times a year	344 (67.5)
	>4 times a year	89 (17.5)
Duration of relationship with rheumatologist (years)		7 (7)
Almost every visit the same rheumatologist, no. (%)		501 (97%)
Trust in physician (CPQ) (range 1–4)		3.48 ± .49
Emotional support of physician (CPQ) (range 1–4)		3.13 ± .49

*Values are the mean ± SD (range) unless otherwise indicated.

SF12 = 12-Item Short Form Health Survey; CPQ = Cologne Preference Questionnaire; PEPPI = Perceived Efficacy in Patient-Provider Interaction; API = Autonomy Preference Index.

### Concordance of preferred and perceived participation

Across decisions, most respondents (59-63%) preferred to share decisions about their treatment with their doctor, though a small but considerable group wanted the doctor to decide (Table 3). We found no significant differences between respondents who had faced the decision versus respondents who had not faced the decision. A small, though statistically significant (Chi-Square = 15.22; df = 6; P = 0.02) difference in the distributions of preferred participation was found between the four decisions: regarding the decision whether or not to start injecting MTX, relatively more respondents preferred to decide by themselves (15% versus 9-11% for the other decisions). The distributions of role preference did not significantly differ between the other three decisions.

**Table 3:** **Preferred and perceived role in medical decision-making**

Decision	Preferred role^1^				Perceived role^1^			
	Doctor (1)	Shared (2)	Patient (3)	Valid N	Doctor (1)	Shared (2)	Patient (3)	Valid N
MDM in general	31%	61%	8%	504	43%	55%	1%	506
Traditional DMARD	32%	59%	10%	491	72%	26%	2%	368
Injecting MTX	25%	60%	15%	466	43%	40%	17%	162
Biologic agent	26%	63%	11%	471	44%	50%	6%	149
Decrease/stop	30%	61%	9%	489	38%	38%	24%	314

*MDM = Medical Decision-making; DMARD = Disease Modifying Anti-Rheumatic Drug; MTX = methotrexate.*

^1^ *Data of perceived role included respondents who had ever faced the decision; data of preferred role included all respondents.*

With regard to the perceived roles, the majority felt that decisions were often made by doctor and patient together (Table 3). Yet, a considerable number of patients felt that ultimately the doctor made the final decision. We found a significant difference between the distributions of perceived participation between the four decisions (Chi-Square = 139.56; df = 6; P < 0.001). Some decisions stand out: 72% of the respondents perceived that the decision to start using a traditional DMARD was made by the doctor alone, as opposed to 38% – 44% for the other decisions. On the other hand, for the decision to start injecting methotrexate or to decrease or stop medication, a considerable number of patients felt they had made the decision by themselves (17% and 24%, respectively).

Table 4 shows the data on concordance of the preferred and perceived roles. For 43% - 62% of the patients, a match was established between the preferred and perceived roles. A considerable group (26% - 54%) perceived “too little” participation, compared to their preference. Again, there was considerable and significant variation between decisions (Chi-Square = 120.99; df = 6; P < 0.001): more than half of the respondents perceived too little participation with the decision to start using a traditional DMARD and almost one third perceived too much participation in deciding to decrease or stop their medication.

**Table 4:** **Concordance between preferred and perceived role**

	Too little participation	Enough participation	Too much participation
MDM in general (n = 496)	29%	61%	10%
Traditional DMARD (n = 330)	54%	43%	4%
Injecting MTX (n = 137)	29%	56%	14%
Biologic agent (n = 129)	30%	62%	8%
Decrease/stop (n = 303)	26%	46%	28%

*MDM = Medical Decision-making; DMARD = Disease Modifying Anti-Rheumatic Drugs; MTX = methotrexate.*

### Satisfaction with the decision process

Most respondents (83% – 89%) felt “satisfied” or “very satisfied” with the decision process in general and for each specific decision. There were however significant differences in satisfaction between the three levels of perceived participation (doctor, shared, patient) (Table 5). For most decisions, patients were more satisfied when they participated in decision-making.

**Table 5:** **Satisfaction with the decision process** ^**1**^ **by perceived role and by concordance**

	Perceived role				Concordance			
	Doctor	Shared	Patient	P^2^	Too little participation	Enough participation	Too much participation	P^3^
	Mean	Mean	Mean		Mean	Mean	Mean	
	(SD)	(SD)	(SD)		(SD)	(SD)	(SD)	
MDM in general (N = 502)	4.0^a^	4.2^a^	4.0	.04*	3.9^ab^	4.2^a^	4.4^b^	.00**
	(0.8)	(0.7)	(0.6)		(0.8)	(0.7)	(0.5)	
	(N = 218)	(N = 278)	(N = 6)		(N = 142)	(N = 302)	(N = 52)	
Traditional DMARD (N = 332)	3.9^a^	4.2^a^	4.3	.00**	3.8^ab^	4.1^a^	4.3^b^	.00**
	(0.7)	(0.6)	(0.5)		(0.6)	(0.6)	(0.7)	
	(N = 234)	(N = 90)	(N = 8)		(N = 177)	(N = 141)	(N = 12)	
Injecting MTX (N = 137)	3.8^a^	4.2^a^	4.0	.02*	3.7^ab^	4.1^a^	4.3^b^	.00**
	(0.9)	(0.6)	(0.7)		(0.8)	(0.7)	(0.7)	
	(N = 53)	(N = 59)	(N = 25)		(N = 41)	(N = 76)	(N = 20)	
Biologic agent (N = 131)	4.0	4.3	4.2	.16	4.1	4.3	3.8	.31
	(0.8)	(0.6)	(1.6)		(0.9)	(0.7)	(1.2)	
	(N = 58)	(N = 67)	(N = 6)		(N = 39)	(N = 80)	(N = 10)	
Decrease/stop (N = 304)	3.9^a^	4.1^ab^	3.8^b^	.00**	3.8^a^	4.1^ab^	3.8^b^	.00**
	(0.8)	(0.6)	(0.8)		(0.8)	(0.7)	(0.8)	
	(N = 115)	(N = 115)	(N = 74)		(N = 80)	(N = 139)	(N = 84)	

*MDM = Medical Decision-making; DMARD = Disease Modifying Anti-Rheumatic Drug; MTX = methotrexate.*

^1^ranging from 1 – 5 in which higher scores indicate more satisfaction.

^2^p-levels for differences between doctor, shared and patient, tested with the Kruskal-Wallis test.

^3^p-levels for differences between too little, enough and too much participation, tested with the Kruskal-Wallis test.

^a or b^Distributions are significant different from each other (post hoc test with Mann Whitney with Bonferroni correction).

*Significant on the .05 level.

**Significant on the .01 level.

Regarding the relationship between satisfaction and concordance, we expected that patients who achieved concordance (“enough participation”) would be more satisfied than those who perceived “too little” or “too much” participation. Our results indeed revealed that, for most decisions, patients who perceived “too little” participation were significantly less satisfied. Yet, getting “too much” participation did not decrease satisfaction. Overall, our results suggest that perceiving “too much participation” is not related to less satisfaction.

### Factors associated with preferred and perceived roles

When analyzing factors associated with the preference for and perception of SDM compared to clinician-led decision-making, only a few significant weak relationships were found in the multivariate binary logistic regression analysis. For the preference for SDM in general medical decision-making only age (OR = 0.960; 95% CI 0.935 - 0.985; p = 0.002) and education (OR = 1.462; 95% CI 1.007 - 2.121; p = 0.046) were significant predictors; meaning that younger and higher educated patients more often prefer SDM than clinician-led decision-making. However, the goodness of fit of the total model was small (Nagelkerke Pseudo R^2^ = 0.102; p = 0.028). No significant relationships were found between preferred role and any of the other variables included in the regression analysis.

For the perception of SDM in general medical decision-making only physical wellbeing (OR = 0.973; 95% CI 0.949 - 0.998; p = 0.036) and emotional support (OR = 2.232; 95% CI 1.172 - 4.251; p = 0.015) were significant predictors; meaning that patients with physical problems and who perceive more emotional support from their attending physician more often perceive SDM than clinician-led decision-making. However again, the goodness of fit of the total model was small (Nagelkerke Pseudo R^2^ = 0.130; p = 0.001). None of the other variables that were included in the regression analysis were significantly related to the perception of SDM.

We also analyzed factors associated with preference for and perception of SDM for specific decisions (data not shown), but no clear pattern arose and relationships were small (R^2^’s for all models < 0.14).

## Discussion

Our study shows that the majority of patients with RA, PsA and AS prefer to share decisions about medication, although a small, but significant group still wants the doctor to decide. These results are in line with other studies in rheumatology [18–21] and other chronic diseases [38].

To the best of our knowledge, our study is the first in the field of rheumatology that examined the concordance between preferred and perceived roles. Our study shows that, in rheumatologic outpatient care, Shared Decision-Making is perceived frequently and patients’ preferences are met in over half of the patients. However, the amount of concordance varied significantly between decisions; a considerable group (on average 34%) still wanted more participation than they perceived. These results are comparable to studies examining other conditions, such as cancer and asthma where concordance levels varying from 34% - 66% have been reported [14, 22, 27, 39–41].

We also examined the relationship between concordance in patient participation and patients’ satisfaction. We expected that patients would be less satisfied with the decision process if they perceived either too little or too much participation. However, our results suggested that patients are only less satisfied if they perceive too little participation. If patients perceived more participation than preferred, they were still highly satisfied. Although many studies have shown that SDM can improve satisfaction [2–6], to our knowledge it has not been previously reported that offering a greater than preferred level of participation is not related to diminished satisfaction, but offering too little is. These findings implicate that patients should be invited to participate in medication decision-making by their rheumatologist at all times.

Previous studies on patient involvement in medical decision-making have mostly looked at decision-making in general. Our study discriminated between medical decision-making in general and four specific decisions that are common in rheumatology. Contrary to our expectations, we found no relevant variety in role preference between these decisions. It seems to be that role preference in decisions about medication for rheumatic diseases is rather stable.

Although we did not find any relevant differences in the distribution of role preferences between the four decisions, we did find differences in the distribution of perceived role and concordance. Two decisions stand out: the decision to start using a traditional DMARD and the decision to decrease or stop a DMARD. With this first decision, the majority of patients (72%) perceived that the doctor decided and in contrast to the other decisions, more than half of the patients (54%) did not achieve their preferred level of participation. An explanation for this finding might be that in the setting of starting a traditional DMARD for the first time there is lack of awareness of choice and too little time for patients to participate. The decision to start a traditional DMARD for the first time is a decision that occurs most commonly in newly diagnosed patients. The current guidelines for early arthritis recommend starting with aggressive treatment as soon as possible, with methotrexate being the recommended drug of first choice. It is plausible that patients initially only receive one treatment recommendation and are not aware of alternative treatment options. Upon receiving the diagnosis, the patient needs to process a lot of information (about the influence of this chronic disease on daily life, starting aggressive treatment, etc.) in a short time. Not being aware of having a choice, little time, and/or an overload of information may be a barrier for patient involvement [11, 12]. In clinical practice, extra time (to think and to create awareness of choice) needs to be considered when dealing with newly diagnosed patients that need to make decisions about starting with a traditional DMARD. Additionally, patients need to be urged to participate to arrive at a decision concordant with their values. These actions may not only enhance patients’ satisfaction, it may also increase patients’ self-efficacy in being adherent to medication use [42–45].

The decision to decrease or stop medication stands out because, in contrast to the other decisions, relatively many respondents (24%) felt they had made this decision by themselves. Moreover, a large group perceived too much participation in this decision. The decision to decrease or stop medication is different from the other decisions, because it occurs when the disease is in remission or when side effects are presented. It is possible that patients are more strongly invited to participate in the decision to decrease or stop their medication, because it is more preference sensitive. Previous studies have shown that patients fear returning symptoms (in the case of remission) or unknown side effects (when changing therapy) [46, 47]. More research is necessary to clarify why patients feel they are too much involved in the decision to decrease or stop medication. These studies should discriminate between decisions to decrease or stop medication in case of remission or to decrease or stop medication when side effects are presented.

The final aim of this study was to examine for each decision which factors were associated with preferred and perceived roles. Although we assessed many possible variables, our results did not reveal any meaningful models to predict preferred or perceived participation in decision-making in general or with reference to specific decisions about DMARDs. In rheumatology, only a few studies have examined associated factors for preferred and perceived roles and those results are inconclusive [18–21, 48]. For example, female gender was significantly associated with higher preferences for involvement in decision-making in one study [20], but not others [18, 19]. Likewise, younger age has been reported as a significant predictor of preference for involvement [18–20], but in our data we only found weak correlations and the results varied per decision. As far as we know, only one previous study has examined associations with perceived involvement in rheumatology [48]. Although this study found several significant associated factors, the presented odds ratios for high involvement were low or with a high confidence interval (indicating a low level of precision of the odds ratio) [49]. Results of studies using other populations were also inconclusive [39, 40, 50–54]. Our findings imply that it remains difficult to identify subgroups that are more in need of being involved. However, as our results revealed that too much participation is not related to diminished satisfaction, we can recommend that caregivers facilitate patient participation in all of their patients. Therefore we suggest training in SDM should be emphasized in educational programs of rheumatologists.

A strength of this study is its large representative sample of patients to examine preferred roles, perceived roles, concordance and satisfaction in various decisions regarding medication use in rheumatology. Due to some limitations of the study, some caution is necessary when interpreting our results. First, due to sizable non-response, our results might be slightly biased. Although we had a response rate of 58%, selection bias might have occurred. It is possible that patients who have no need of participating in medical decision-making, are less interested in (responding to questionnaires about) patient participation. Second, due to limited resources we chose to conduct a retrospective study and therefore it is possible that recall bias occurred. We questioned patients about the first time they faced these decisions. Some of these decisions may have occurred years before the study. Even though we did not find any significant differences in preferred and perceived role between patients with a long (>1 year) and short (<1 year) illness duration, the limitation of possible recall bias remains. We therefore recommend a prospective study which questions patients at the time of the decision. Third, no patient representatives were included in our research group as has been recently recommended [55]. Patient representatives can provide valuable suggestions about which aspects to include in the questionnaires and the interpretation of results. Yet, as the current study is part of a larger project to develop a Patient Decision Aid (PtDA) for anti-rheumatic drugs in which patients were repeatedly and extensively involved in various research and design activities, we feel that we have included patient perspectives to at least some extend.

## Conclusions

In conclusion our study shows that arthritis patients appreciate being involved in decisions about their medication and that shared decision-making is perceived frequently in rheumatology outpatient care. Yet, patient participation can be further improved, particularly in decision-making about starting a traditional DMARD for the first time. As our results revealed that too much participation is not related to diminished satisfaction, we recommend assessing patients’ preferred and perceived role in medical decision-making regularly and invite patients to participate in every decision. Moreover, we recommend rheumatologists and other caregivers to consider extra time for patients to create awareness of choice and to process all the information, especially when dealing with newly diagnosed patients that need to make decisions about initiating a traditional DMARD.
